# ERG mediates the differentiation of hepatic progenitor cells towards immunosuppressive PDGFRα^+^ cancer-associated fibroblasts during hepatocarcinogenesis

**DOI:** 10.1038/s41419-024-07270-9

**Published:** 2025-01-18

**Authors:** Haoran Bai, Xinyu Zhu, Lu Gao, Shiyao Feng, Hegen Li, Xiaoqiang Gu, Jiahua Xu, Chen Zong, Xiaojuan Hou, Xue Yang, Jinghua Jiang, Qiudong Zhao, Lixin Wei, Li Zhang, Zhipeng Han, Wenting Liu, Jianxin Qian

**Affiliations:** 1 Department of Oncology, Longhua Hospital Affiliated to Shanghai University of Traditional Chinese Medicine, Shanghai, China; 2 Changhai Clinical Research Unit, Changhai Hospital of Naval Medical University, Shanghai, China; 3 National Center for Liver Cancer, Shanghai, China; 4 Department of Urology, Chaohu Hospital of Anhui Medical University, HeFei, Anhui China; 5 Department of Medical Oncology, Fudan University Shanghai Cancer Center, Shanghai, China

**Keywords:** Cancer microenvironment, Tumour biomarkers

## Abstract

Cancer-associated fibroblasts (CAFs) play important roles in the occurrence and development of hepatocellular carcinoma (HCC) and are a key component of the immunosuppressive microenvironment. However, the origin of CAFs has not been fully elucidated. We employed single-cell sequencing technology to identify the dynamic changes in different subsets of fibroblasts at different time points in rat primary HCC model. Inflammation-associated CAFs (*Pdgfrα*^+^ CAFs) were subsequently identified, which demonstrated a significant correlation with the survival duration of HCC patients and a dual role in the tumour microenvironment (TME). On the one hand, they secrete the chemokines CCL3 and CXCL12, which recruit macrophages to the tumour site. On the other hand, they produce TGFβ, inducing the polarization of these macrophages towards an immunosuppressive phenotype. According to the in vitro and in vivo results, hepatic progenitor cells (HPCs) can aberrantly differentiate into PDGFRα^+^ CAFs upon stimulation with inflammatory cytokine. This differentiation is mediated by the activation of the MAPK signaling pathway and the downstream transcription factor ERG via the TLR4 receptor. Downregulating the expression of ERG in HPCs significantly reduces the number of PDGFRα^+^ CAFs and the infiltration of tumour-associated macrophages in HCC, thereby suppressing hepatocarcinogenesis. Collectively, our findings elucidate the distinct biological functions of PDGFRα^+^ cancer-associated fibroblasts (PDGFRα^+^ CAFs) within the TME. These insights contribute to our understanding of the mechanisms underlying the establishment of an immunosuppressive microenvironment in HCC, paving the way for the exploration of novel immunotherapeutic strategies tailored for HCC treatment.

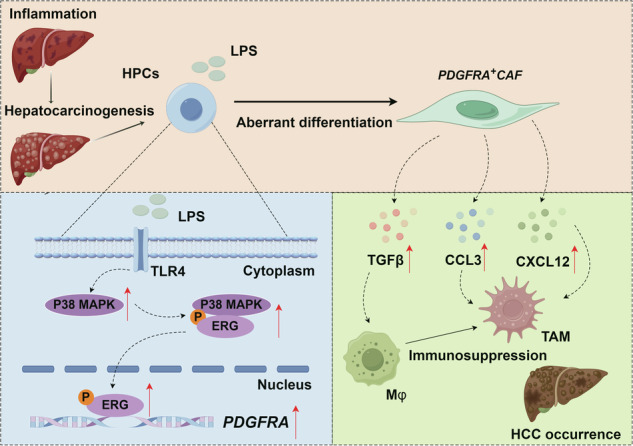

## Introduction

The immunosuppressive microenvironment (ISME) is a distinctive state within the tumour microenvironment (TME), characterized by its ability to suppress the host antitumour immune response, thereby facilitating the growth and metastasis of cancer [1, 2]. This microenvironment is composed of various components, including tumour cells, immune cells, stromal cells (such as cancer-associated fibroblasts (CAFs)), the extracellular matrix, and a variety of soluble molecules. Within the ISME, tumour cells or stromal cells can manipulate the immune system to avoid detection, while also recruiting and activating diverse immunosuppressive cells, such as myeloid-derived suppressor cells, tumour-associated macrophages (TAMs), and T cells. The ISME is intricately linked to hepatocellular carcinoma (HCC), exerting a profound effects on tumour initiation, progression, and response to therapy [3]. Characterized by high heterogeneity, the immune landscape of HCC includes a spectrum of immunosuppressive cell types, with CAFs being particularly notable [4, 5]. Moreover, the ISME has consistently been a significant reason for patients’ poor response to first-line medications such as immune checkpoint inhibitors [6]. However, the specific mechanisms through which the ISME forms in HCC remain unclear.

CAF populations are diverse and dynamic and originate from various cell sources, which contributes to their functional diversity. Su et al. reported that CD10^+^GPR77^+^ CAFs promote tumour formation and chemoresistance by providing a survival niche for cancer cells [7]; Ye et al. reported that myofibroblast CAFs secrete an extracellular matrix that specifically limits the natural killer secreted extracellular matrix and natural killer (NK) cell cytotoxicity to promote tumour growth [8]. However, the complex interactions among CAFs, HCC cells, and immune cells, especially across different CAF subtypes, are not yet fully understood. A comprehensive understanding of the multidimensional dynamics between CAFs and the immune cells that infiltrate the TME is essential for deciphering the mechanisms by which CAFs contribute to immunosuppression. Recent methodological advancements, including single-cell sequencing and flow cytometry, have facilitated the precise characterization and functional analysis of CAF subtypes across various cancer types. Recent studies have provided evidence for the existence of procancer CAFs. Procancer CAFs, including myofibroblastic CAFs and inflammatory CAFs, are involved in extracellular matrix (ECM) remodelling, tumour-promoting inflammation, and the ability of the immune microenvironment to be modulated towards immunosuppression. Overall, various CAF subtypes may activate distinct molecular pathways to influence the TME and participate in the construction of the ISME [9–11]. Therefore, elucidating the heterogeneity and the specific traits of CAF subpopulations is essential for the development of therapeutic strategies aimed at either targeting protumorigenic CAFs or enhancing the efficacy of immunological therapies.

In our study, we utilized single-cell RNA (scRNA) sequencing to delineate the heterogeneity and temporal dynamics of CAF subgroups in a diethylnitrosamine (DEN)-induced primary HCC model. Furthermore, our findings were corroborated by cross-validating them with human single-cell RNA datasets. We identified a subset of *Pdgfrα*^+^ CAFs, characterized as inflammatory CAFs (iCAFs), within the TME. iCAFs demonstrated significant tumour infiltration and a robust positive correlation with the survival duration of HCC patients. Furthermore, these iCAFs facilitated the recruitment of macrophages into the TME and induced their polarization towards an immunosuppressive phenotype. We observed that PDGFRα^+^ CAFs may originate from hepatic progenitor cells (HPCs), suggesting that HPC-derived iCAFs may contribute to the establishment of an immunosuppressive microenvironment during hepatocarcinogenesis. Finally, we elucidated the molecular mechanisms underlying the aberrant differentiation of HPCs into PDGFRα^+^ CAFs, which could provide insights into the potential mechanisms involved in the formation of the ISME.

## Results

### Identification of CAF subpopulations derived during hepatocarcinogenesis by single-cell RNA sequencing during hepatocarcinogenesis

**Fig. 1:**
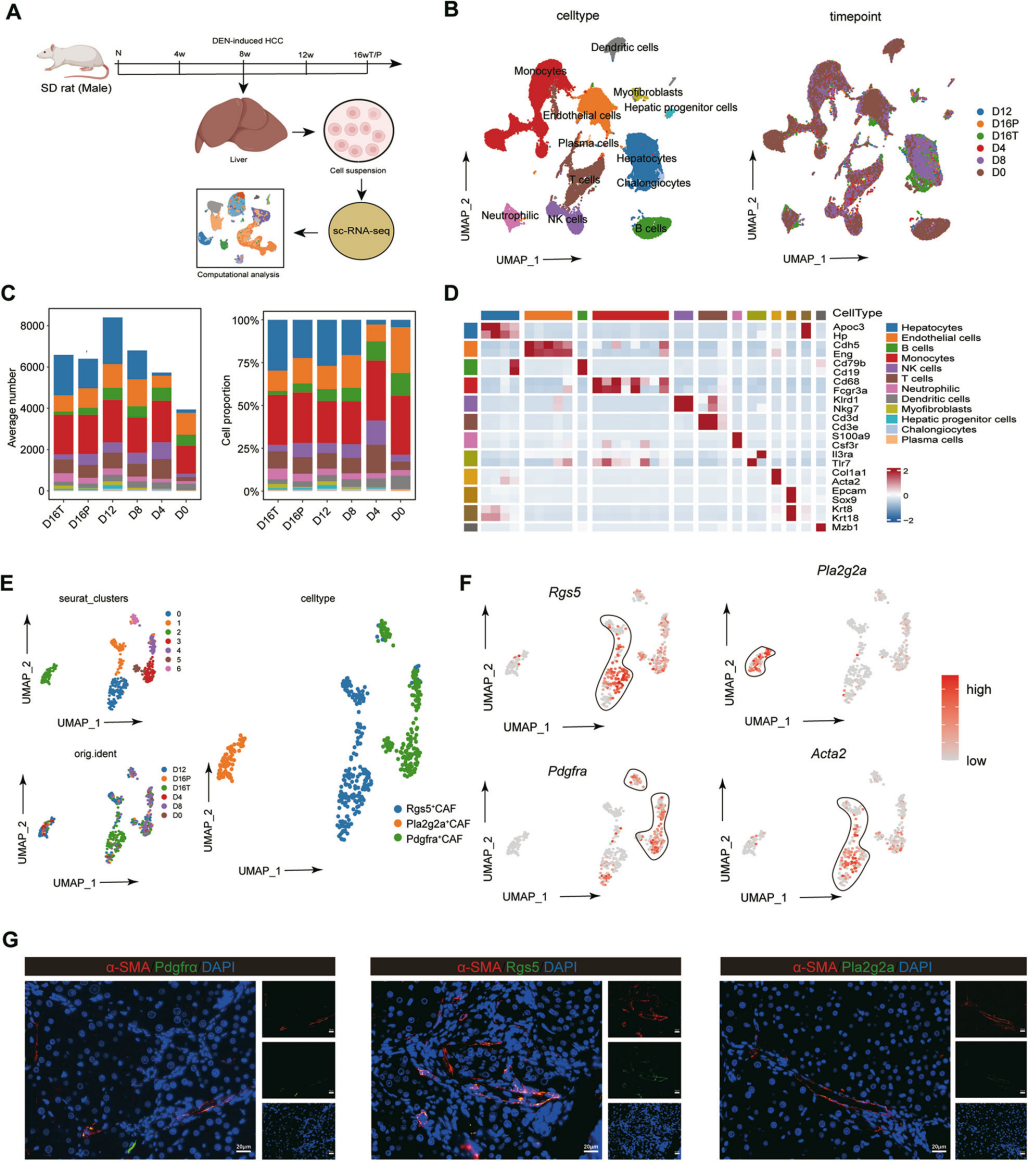
Identification of CAF subpopulations during hepatocarcinogenesis by single-cell RNA sequencing. **A** Schematic representation of the experimental strategy. **B** UMAP plot showing the clustering results for single cells from the livers of rats exposed to DEN among all cell types at different times: cholangiocytes, HPCs, endothelial cells, natural killer cells, T cells, B cells, plasma cells, dendritic cells, neutrophils, myofibroblasts, monocytes and hepatocytes. The different time points and cell types are indicated by different colours. Schematic of the schedule for the collection and processing of liver tissue for single-cell RNA sequencing. D0, liver tissue from rats exposed to DEN for 0 weeks; D4, liver tissue from rats exposed to DEN for 4 weeks; D8, liver tissue from rats exposed to DEN for 8 weeks; D12, liver tissue from rats exposed to DEN for 12 weeks; D16T, tumour tissue from rats exposed to DEN for 16 weeks; D16P, peritumour tissue from rats exposed to DEN for 16 weeks. **C** The number and proportion of the cell types in the liver tissue of rats exposed to DEN for different durations. **D** Heatmap showing DEGs among different cell types. **E** UMAP plot showing CAFs coloured by clusters (upper left panel), time of exposure to DEN (lower left panel) and subgroup markers(right panel). **F** UMAP plot showing the expression of typical markers (*Rgs5, Pla2g2a, Pdgfra, Acta2*) in the CAF subgroups. **G** IF staining confirmed the existence of three CAF subgroups (*n* = 15). The scale bar represents 20 μm.

As a method to delineate the CAF landscape during hepatocellular carcinoma (HCC), we utilized single-cell RNA (scRNA) sequencing to analyse liver tissues from a DEN-induced rat HCC model, whose pathological progression closely mirrors that of human liver cancer. Our previous studies has already evaluated the liver injury status of DEN-pretreated rats at 0 weeks (N), 4 weeks (D4), 8 weeks (D8), 12 weeks (D12), and 16 weeks (D16). DEN-induced model rats developed HCC at D12 to D16. By the end of D16, most of the rats in the study presented visible tumours (> 1 mm) in the livers [12]. Therefore, liver tissues from DEN-induced model rats were collected at different time points (N, D4, D8, D12, and D16) and scRNA sequencing was performed. After stringent quality control and removal of the batch effect, we obtained a final collection of 41,889 cells for computer analysis (Figs. 1A and S1A–D). Unsupervised clustering analysis identified 30 clusters which were projected using the uniform manifold approximation and projection (UMAP) algorithm. The UMAP representation of different samples revealed a similar distribution of single cells when each sample was analysed. According to expression of marker genes, 12 major distinct cell types were identified including cholangiocytes, hepatic progenitor cells (HPCs), natural killer (NK) cells, T cells, B cells, plasma cells, dendritic cells (DCs), neutrophils, mesenchymal stem cells (MSCs), endothelial cells (ECs), monocytes and hepatocytes (Fig. 1B). The proportions and average numbers of the distinct cell types in different liver samples are shown in Fig. 1C. Differential expression analyses revealed cluster-specific transcriptomic profiles (Fig. 1D). The CAFs from the 6 samples were subsequently clustered into 6 subgroups according to distinct CAF transcriptomic signatures. Six CAF clusters were identified as *Rgs5*^+^ CAFs, *Pla2g2a*^+^ CAFs, and *Pdgfra*^+^ CAFs (Fig. 1E). *Rgs5*^+^ CAFs are RGS5 positive and characterized by high expression of collagen and EMT- associated genes, which might be associated with the production of the ECM and the formation of tumour nodular tissue. *Pla2g2a*^+^ CAFs expressed high levels of PLA2G2A and metabolism-related genes, which indicated that *Pla2g2a*^+^ CAFs might be involved in several metabolic processes. *Pdgfrα*^+^ CAFs presented high levels of PDGFRα and inflammation-associated genes, which might play important roles in the inflamed tumour microenvironment (Figs. 1F and S1E, F). Liver tissues were collected from D12 rats for multiplex immunofluorescence staining to verify the existence of *Rgs5*^+^ CAFs, *Pla2g2a*^+^ CAFs and *Pdgfra*^+^ CAFs. The results confirmed the presence of *RGS5*^+^ CAFs, *PLA2G2A*^+^ CAFs and *PDGFRA*^+^ CAFs in the periportal vein and fibrotic areas (Fig. 1G). Therefore, we revealed that the RGS5^+^ CAFs, PLA2G2A^+^ CAFs and PDGFRα^+^ CAFs are closely related to hepatocarcinogenesis during the progression of HCC.

### PDGFRα^+^CAFs construct an immunosuppressive microenvironment in HCC by producing immunosuppressive cytokines and recruiting TAMs

**Fig. 2:**
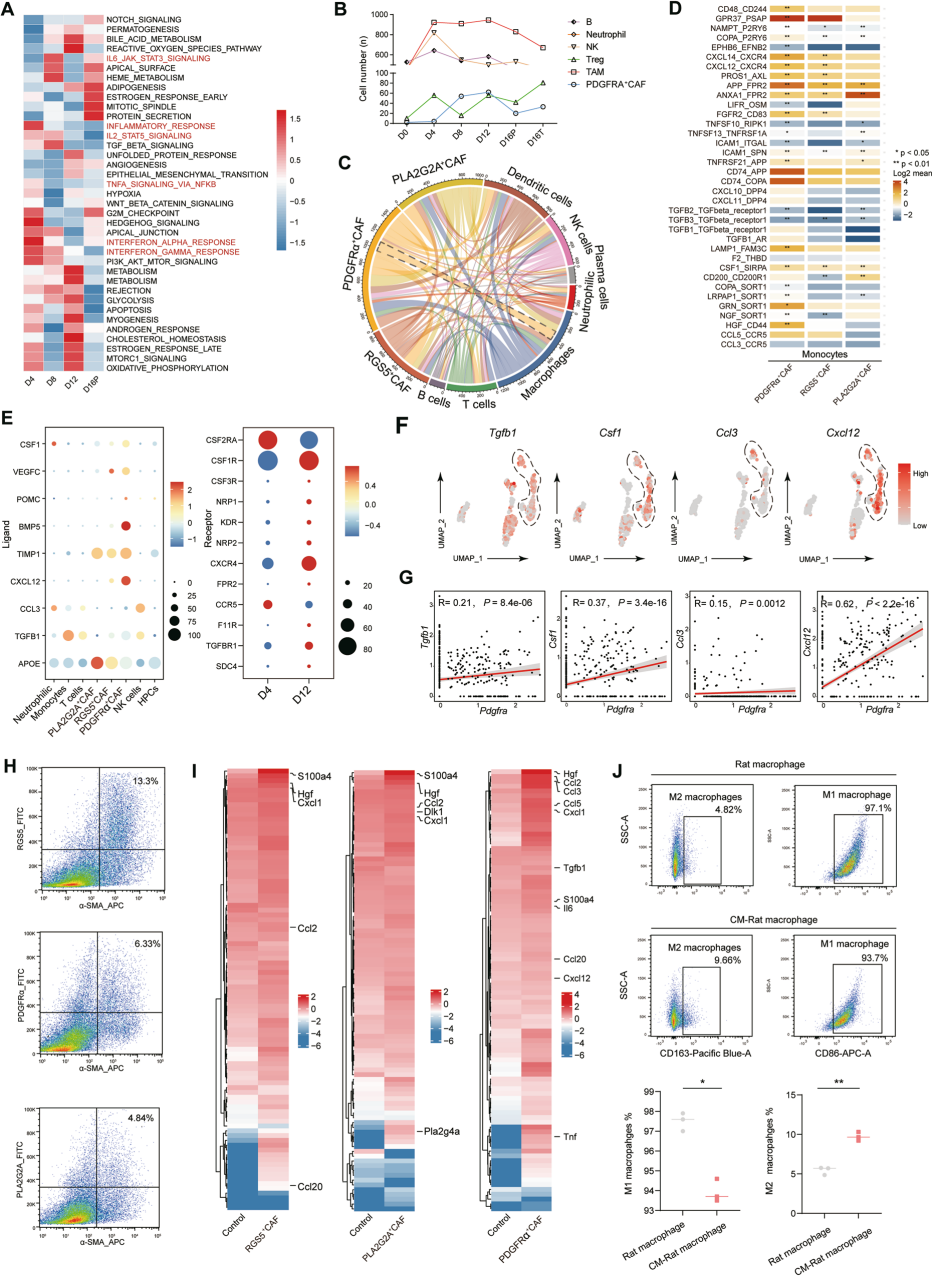
PDGFRα^+^ CAFs construct an immunosuppressive microenvironment in HCC by producing immunosuppressive cytokines and recruiting TAMs. **A** Heatmap showing differences in pathway activities per cell scored by GSVA across different durations of exposure to DEN. **B** The numbers of myeloid-derived cells and iCAFs (*Pdgfra*^+^ CAFs) according to different durations of exposure to DEN. **C** Heatmap showing L-R enrichment analysis results for CAFs and other cell types, with the fewest L-R pairs represented as blue boxes and greatest number of L-R pairs represented as red boxes. **D** Heatmap showing the ligand-receptor pairs of cytokines between CAFs and monocytes. **E** Dot plots showing the expression levels of ligands in different cell types (left panel) and receptors as in different times after exposure to DEN (right panel). **F** UMAP plot showing the expression levels of *Tgfb1, Csf1, Ccl3* and *Cxcl12* in *Pdgfra*^+^CAFs. **G** Correlations between PDGFRα^+^ CAFs and the associated cytokines in TCGA-LIHC cohort. **H** The frequency of CAF subsets from liver tissues of rats exposed to DEN for 12 weeks were examined by flow cytometry. **I** Heatmap showing protein levels in the supernatants secreted by CAFs, as measured by Olink^®^. **J** Rat macrophages identified by flow cytometry after co-cultured with supernatant separately from the CAF subgroups for 24 h.

We used gene set variation analysis to observe the dynamic changes in inflammation levels and explore the function of *Pdgrfa*^+^ CAFs in the TME in the rat primary HCC model. As shown in Fig. 2A, from D4 to D8 of HCC induction, inflammation-associated genes were expressed at high levels in the liver. However, beginning at D12 after the occurrence of HCC, the levels of inflammation-associated gene sets were significantly reduced. We also observed that the temporal abundance curves of *Pdgfra*^+^ CAFs and TAMs from N to D16 presented the highest degree of similarity (Fig. 2B). We subsequently employed CellPhoneDB to examine the cross-talk between *Rgs5*^*+*^ CAFs, *Pla2g2a*^*+*^ CAFs, and *Pdgfra*^*+*^ CAFs and the other immune cells. We found that the strongest occurred between CAFs and monocytes (Fig. 2C). Moreover, compared with other fibroblast subsets, *Pdgfra*^*+*^ CAFs were enriched in chemokine and inflammation-related receptor‒ligand, particularly in their associations with TAMs (Fig. 2D). Therefore, we hypothesized that *Pdgfra*^*+*^ CAFs may play a role in modulating macrophages within the TME. We explored how *Pdgfra*^*+*^ CAFs regulate macrophages, by further investigating the expression of coinhibitory and chemokine-related ligands and receptors via a ligand‒receptor interaction analysis. The results indicated that the high expression of ligands such as *Tgfb1*, *Csf1*, *Ccl3*, and *Cxcl12* in *Pdgfra*^*+*^ CAFs corresponded with the upregulation of their respective receptors on D12 monocytes at D12 (Fig. 2E–G). Next, fluorescence-activated cell sorting (FACS) was used to sort *Rgs5*^+^ CAFs, *Plag2a*^+^ CAFs and *Pdgfra*^*+*^ CAFs from D12 tumour tissue, and conditioned medium was collected to perform Olink detection. Consistent with the results of the single cell sequencing analysis, significantly higher expression of TGF-β, CCL3 and CXCL12 was observed in conditioned medium from PDGFRα^*+*^ CAFs (Fig. 2H–I). We subsequently cultured rat macrophages (NR8383) with the supernatant from PDGFRα^*+*^ CAFs for 48 h. The flow cytometry analysis revealed that PDGFRα^*+*^ CAFs can partially induce the differentiation of macrophages into anti-inflammatory M2 macrophages. Taken together, these results indicate that PDGFRα^*+*^ CAFs might construct an immunosuppressive microenvironment in HCC by producing immunosuppressive cytokines, recruiting monocytes and inducing the polarization of monocytes towards anti-inflammatory M2 macrophages (Fig. 2J).

### Large numbers of PDGFRα^+^CAFs infiltrate tumour tissues and are closely associated with a poor prognosis for HCC patients

**Fig. 3:**
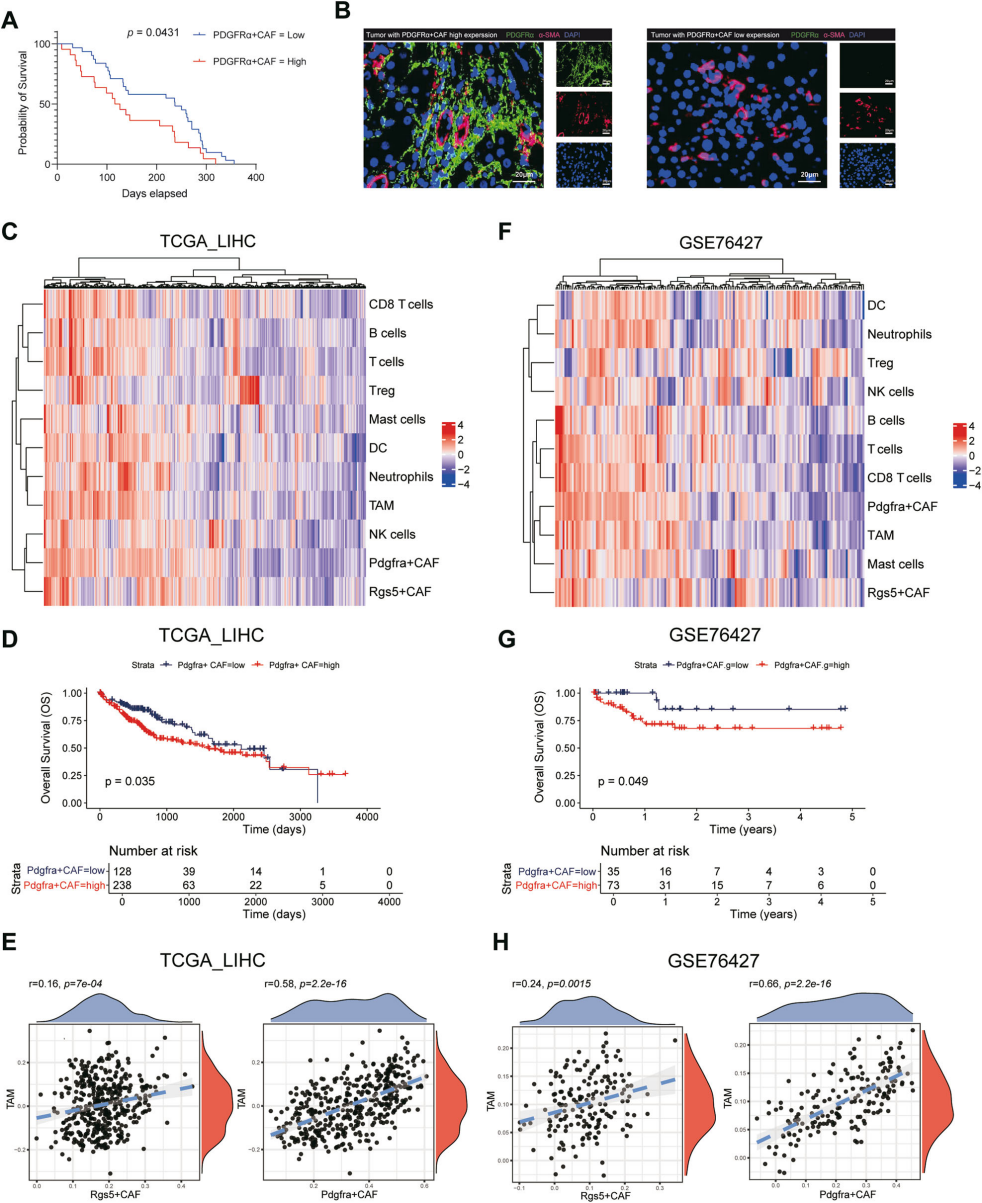
Correlation between PDGFRα^+^ CAFs and the prognosis of HCC patients. **A** Kaplan–Meier overall survival curves of tumour tissues from 58 patients with HCC by co-expression of PDGFRα and α-SMA in multi-color staining. *p* value was determined by Kaplan-Meier survival curves and log-rank test. **B** PDGFRα and α-SMA co-expression was detected in tumor tissues from 58 patients with HCC by multi-color staining. Scale bars represents 20 μm. **C** Heatmap showing the cell abundance predicted per sample from TCGA-LIHC cohort by CIBERSORTx. The row z scores are shown. **D** Kaplan–Meier overall survival curves of tumour tissues from TCGA-LIHC cohort with HCC stratified by by the level of PDGFRα^+^ CAFs. The *p* value was determined from Kaplan-Meier survival curves and the log-rank test. **E** Correlations between CAFs and different macrophages in TCGA-LIHC cohort. The coefficient was calculated via Spearman’s correlation analysis. **F** Heatmap of cell abundance predicted per sample from the GSE76247 HCC cohort by CIBERSORTx. The row z scores are shown. **G** Kaplan–Meier overall survival curves of tumour tissues from the GSE76247 HCC cohort stratified by the levels of PDGFRα^+^ CAFs. The *p* value was determined from Kaplan-Meier survival curves and the log-rank test. **H** Correlations between CAFs and different macrophages in the GSE76247 HCC cohort. The coefficient was calculated via Spearman’s correlation analysis.

We examined the expression of PDGFRα and α-SMA in HPCs from adjacent nontumor tissues from clinical patients and divided the patients into PDGFRα^*high*^/α-SMA^*high*^ and PDGFRα^*low*^ /α-SMA^*high*^ groups (Fig. 3B and Table 1).The recurrence analysis revealed that the PDGFRα^*high*^ /α-SMA^*high*^ group had a shorter recurrence time than the PDGFRα^*low*^ /α-SMA^*high*^ group(Fig. 3A), which suggested that the expression of PDGFRα was correlated with the malignant transformation of HPCs into iCAFs and the hepatocarcinogenesis. We further analysed HCC clinical data to understand the clinical relevance of PDGFRα^+^ CAFs in relation to liver cancer patients. A single-sample gene set enrichment analysis approach was performed to to assess the infiltration of PDGFRα^+^ CAFs in HCC patients. The results from TCGA_LIHC and GEO databases (GSE76427) revealed that compared with RGS5^+^ CAFs, substantial infiltration of TAMs and PDGFRα^+^ CAFs was observed in tumours. (Fig. 3F, G). Furthermore, we categorized patients from TCGA_LIHC and GSE76427 into two groups based on high and low levels of PDGFRα^+^ CAFs. The survival analysis revealed that patients with high levels of PDGFRα^+^ CAFs had poorer prognoses than patients with low expression (Fig. 3C, D). The correlation analysis also revealed a positive correlation among RGS5^+^ CAFs, PDGFRα^+^ CAFs and macrophages, suggesting a potential regulatory effect of CAFs on macrophages. (Fig. 3E, H). These results suggest that PDGFRα^+^ CAFs also play an important role in HCC patients.

**Table 1:** Demographic and baseline characteristics of 58 HCC patients.

Factors	Value	Percentage (%)
Age	53 ± 8.71	
Gender (male/female)	45/13	77.6/22.4
HBsAg (positive)	54/4	93.1/6.9
Cirrhosis	50/8	86.2/13.8
PDGFRα^high^/α-SMA^high^ expression	29	50.0
PDGFRα^low^/α-SMA^high^ expression	27	46.6

### Lipopolysaccharide induces the differentiation of HPCs towards PDGFRα^+^ CAFs

**Fig. 4:**
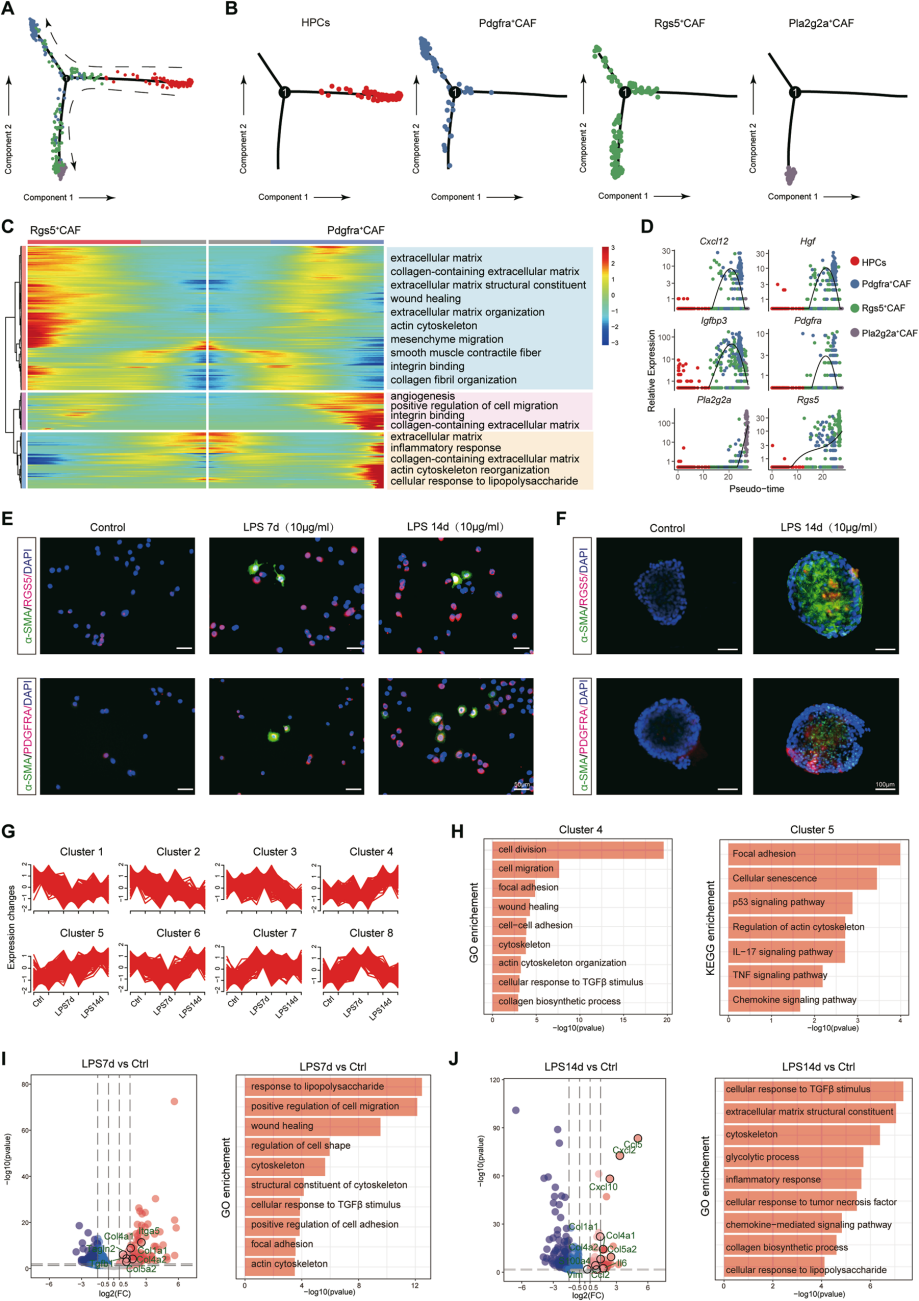
Lipopolysaccharide induces the differentiation of HPCs towards PDGFRα^+^ CAFs. **A**, **B** Pseudotime trajectories of all CAFs revealing 3 branches. The blue, red, green and grey circles indicate cells at the termini of the branches. **C** Heatmap showing dynamic changes in gene expression in the CAF lineage. The genes were clustered into 3 gene sets, each of which was characterized by specific expression profiles, as depicted by a selection of genes characteristic of each cluster. Rows: GO enrichment analysis of DEGs; columns: cells shown in pseudotime order. **D** Expression of key regulators plotted along the pseudotime axis. **E**, **F** The expression of CAF markers (α-SMA, RGS5, and PDGFRα) was detected in HPCs treated with LPS for 7 days and 14 days by immunofluorescence staining. Nuclei were stained with DAPI (blue). **G** Fuzzy Cmeans clustering identified 8 distinct temporal patterns of gene expression. The *x-*axis shows three stages of HPCs (control and treated with LPS for 7 days and 14 days), whereas the *y*-axis shows the log2-transformed, normalized intensity ratios at each stage. (**H** Bar graphs showing the pathways identified after KEGG and GO enrichment analysis of genes in Cluster 4 and 5, respectively. **I**, **J** Volcano plot showing differentially expressed genes (DEGs) between HPCs treated with LPS for 7 days and untreated HPCs (**I**, left panel) or between HPCs treated with LPS for 14 days and untreated HPCs. (**J** left panel). The bar graphs show the Gene Ontology (GO) functional enrichment analysis results for the DEGs between the group treated with LPS for 7 days and the control group and between the group treated with LPS for 14 days and the control group.

In our previous study, we reported that LPS can induce HPCs to differentiate into myofibroblasts and that HPC-derived myofibroblasts create a tumour microenvironment and contribute to the proliferation and malignant transformation of HPCs, which eventually leads to hepatocarcinogenesis [12]. HPCs normally reside in biliary ducts and can be activated by chronic liver damage [13, 14]. During acute liver injury, normal hepatocytes repair liver injury by proliferation and an increased cell volume [15]. However, the proliferative potential of liver cells is exhausted during chronic liver injury and HPCs are activated to repair liver injury through hepatotropic and biliary differentiation [16]. Therefore, we further observed the relationships among CAFs and HPCs from 0 to 12 weeks (0, 4, 8 and 12 weeks) by constructing a transcriptional trajectory of these cells on a pseudotime scale using Monocle. The results indicated that HPCs were distributed at one end of the pseudotime trajectory, *Pdgfra*^+^ CAFs and *Pla2g2a*^+^ CAFs were distributed at the end of the other two pseudotime trajectory and *Rgs5*^+^ CAFs were distributed at the all three pseudotime trajectory at the same time, which suggesting that the three subgroups of CAFs (*Pdgfra*^+^ CAFs, *Pla2g2a*^+^ CAFs and *Rgs5*^+^ CAFs) might all be derived from HPCs (Fig. 4A). Furthermore, as shown in Fig. 4B, HPCs might differentiate into *Rgs5*^+^ CAFs first, and *Pdgfra*^+^ CAFs and *Pla2g2a*^+^ CAFs might be derived from *Rgs5*^+^CAFs. To investigate the change of expression profile between *Rgs5*^+^ CAFs and *Pdgfra*^+^ CAFs at the pseudotime trajectory, GO enrichment analyses of BP terms were performed on the differentially expressed genes (DEGs) to investigate the changes in the expression profiles between *Rgs5*^+^ CAFs and *Pdgfra*^*+*^ CAFs in the pseudotime trajectory. The results revealed that *Rgs5*^+^ CAFs were enriched mainly in functions related to the extracellular matrix, collagen-containing extracellular matrix, extracellular matrix structural constituent, wound healing, extracellular matrix organization, actin cytoskeleton, mesenchyme migration, smooth muscle contractile fibre, integrin binding and collagen fibril organization wheres *Pdgfra*^+^ CAFs were enriched mainly in functions related to angiogenesis, positive regulation of cell migration, integrin binding, the collagen-containing extracellular matrix, the extracellular matrix, the inflammatory response, the collagen-containing extracellular matrix, actin cytoskeleton reorganization and the cellular response to lipopolysaccharide (Fig. 4C). We also detected the expression of typical genes (*Cxcl12, Hgf, lgfbp3 and Pdgfra*) at the branch of *Rgs5*^+^ CAFs to *Pdgfra*^+^ CAFs in the pseudotime trajectory (Fig. 4D). We treated HPCs with LPS for 7 days and 14 days to validate the differentiation of HPCs into CAFs. In vitro differentiation experiments confirmed that short-term (7 days) LPS treatment notably promoted the expression of the myoCAF marker RGS5, whereas long-term (14 days) LPS treatment promoted the expression of the iCAFs marker PDGFRα (Fig. 4E, F). We successfully used single-cell sequencing technology to capture HPCs from a rat HCC model and then observed changes in gene expression in HPCs at different time points during hepatocarcinogenesis. We applied the fuzzy C-means algorithm to cluster the transcript expression profiles across all the developmental stages. In total, we observed eight distinct clusters of temporal expression patterns. Among them, Clusters 4 and 5 contained genes whose expression was upregulated, and a KEGG enrichment analysis was subsequently performed (Fig. 4G). The genes in Cluster 4 were enriched in the following Gene Ontology (GO) terms: cell division, cell migration, focal adhesion, wound healing, cell-cell adhesion, cytoskeleton, actin cytoskeleton organization, cellular response to TGFβ stimulus, and the collagen biosynthetic process. The genes in Cluster 5 were enriched in functions related to the following KEGG pathways: focal adhesion, cellular senescence, the p53 signalling pathway, regulation of the actin cytoskeleton, the IL-17 signalling pathway, the TNF signalling pathway, and the chemokine signalling pathway (Fig. 4H). GO and KEGG enrichment analyses were performed on the differentially expressed genes (DEGs) and proteins between different time points to investigate the changes in the expression profiles of HPCs during hepatocarcinogenesis. The gene expression profile of HPCs was analyzed at 0 days, 7 days and 14 days. Compared with HPCs collected at 0 days, the GO analysis revealed that the upregulated genes at the 7 days were enriched mainly in functions related to the response to lipopolysaccharide, positive regulation of cell migration, wound healing, regulation of cell shape, cytoskeleton and structural constituent of the cytoskeleton (Fig. 4I). Compared with HPCs collected at the 0 days, the GO analysis revealed that the upregulated genes at the 14 days were enriched mainly in functions related to the response to the cellular response to TGFβ stimulus, extracellular matrix structural constituents, the cytoskeleton, glycolytic processes and the inflammatory response (Fig. 4J). These results indicated that in the tumour microenvironment, HPCs induced by LPS might differentiate into Rgs5^+^ CAFs and then be transformed into PDGFRα^+^ CAFs.

### The transcription factor ERG plays a key role in mediating the differentiation of HPCs towards PDGFRα ^+^ CAFs

**Fig. 5:**
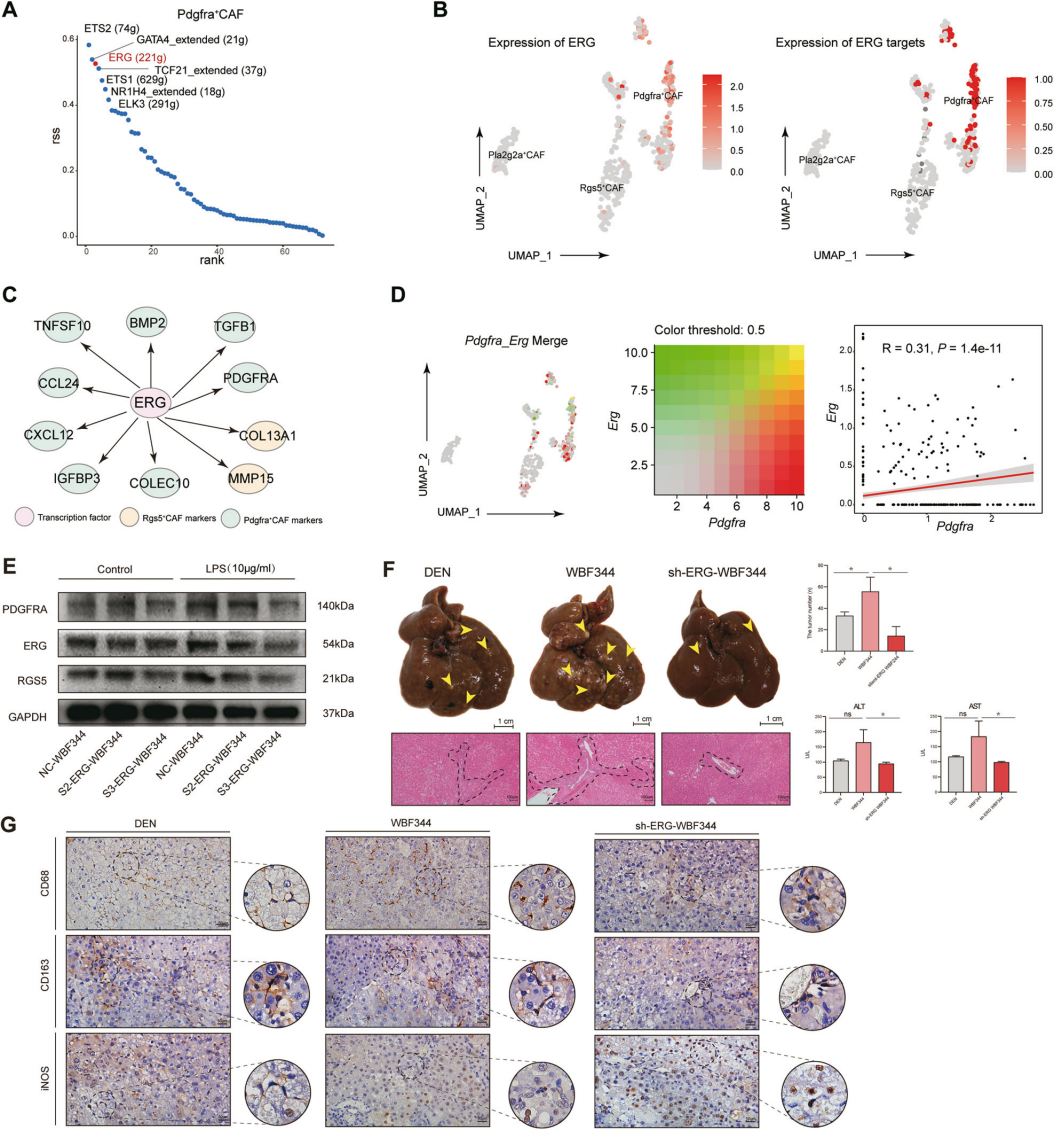
ERG played a key role in mediating the differentiation of HPCs towards PDGFRα^+^ CAFs. **A** Dot plot showing the top 8 regulons in *Pdgfra*^*+*^ CAFs, which are labelled on the plot. The specificity score is shown on the y-axis. **B** UMAP plot showing the expression level of the transcription factor ERG in the regulon in *Pdgfra*^*+*^ CAFs (left side). UMAP plot showing the expression of the ERG target in *Pdgfra*^*+*^ CAFs (right side). **C** The collection of all target genes of the transcription factor ERG in *Pdgfra*^*+*^ CAFs and *RGS5*^*+*^ CAFs predicted by SCENIC. **D** UMAP plot showing the levels of *Erg* and the target gene *Pdgfra* (left panel), and the correlation between the transcription factor ERG and the target gene *Pdgfrα* (right panel). **E** Compared with untreated WBF344 cells, the expression of ERG in LPS-treated WBF344 cells was inhibited by the shRNA and the levels of ERG, PDGFRα and RGS5 were examined in each group by western blotting. **F** Rats (*n* = 6, respectively) treated with DEN for 8 weeks received a 200 μl injection of WBF344 cells or WBF344 cells in which *Erg* was inhibited by an shRNA (1 × 10^6^ cells in PBS) into the tail vein three times per week, and HCC occurrence was observed in each group to verify the tumorigenic potential of ERG. The tumours were observed via microscopy. The number of tumours was calculated. Serum levels of AST and ALT were determined to indicate the extent of liver damage. The data are presented as the means ± SDs. *p < 0.05; ns, not significant. **G** CD68, CD163 and iNOS expression were detected by IHC in the different groups. The number of positive cells was calculated in each group was calculated, and the data are presented as the means ± SDs. *p < 0.05.

We performed SCENIC analysis to investigate transcription factors in HPCs from the scRNA sequencing data. The results indicated that the transcription factor ERG was expressed in HPCs from a rat model, and SCENIC also revealed a network of ERG in HPCs (Fig. 5A–C). ERG was highly expressed in *Pdgfra*^+^ CAFs, and ERG expression was positively correlated with the level of *Pdgfra*. (Fig. 5D). An HPC cell line (WBF344) was transfected with a lentivirus containing the *Erg* shRNA to investigate the mechanism involved in the ERG-induced aberrant differentiation of HPCs. The *Erg* shRNA effectively reduced the ERG expression in HPCs (Fig. S1H, Supporting Information). Knocking down *Erg* in HPCs strongly inhibited the aberrant differentiation of HPCs into CAFs (Figs. 5E and S1F from Supporting Information). We further investigated the effect of inhibiting ERG expression on the tumorigenicity of HPCs. As shown in Fig. 5F, the tumorigenic potential of HPCs was suppressed by knocking down the level of *Erg*. Subsequent in vivo experiments revealed that knocking down *Erg* expression significantly inhibited the recruitment and polarization of M2 macrophages (Fig. 5G).

### The TLR4-P38 MAPK signalling pathway contributes to the LPS-induced differentiation of HPCs into PDGFRα^+^ CAFs

**Fig. 6:**
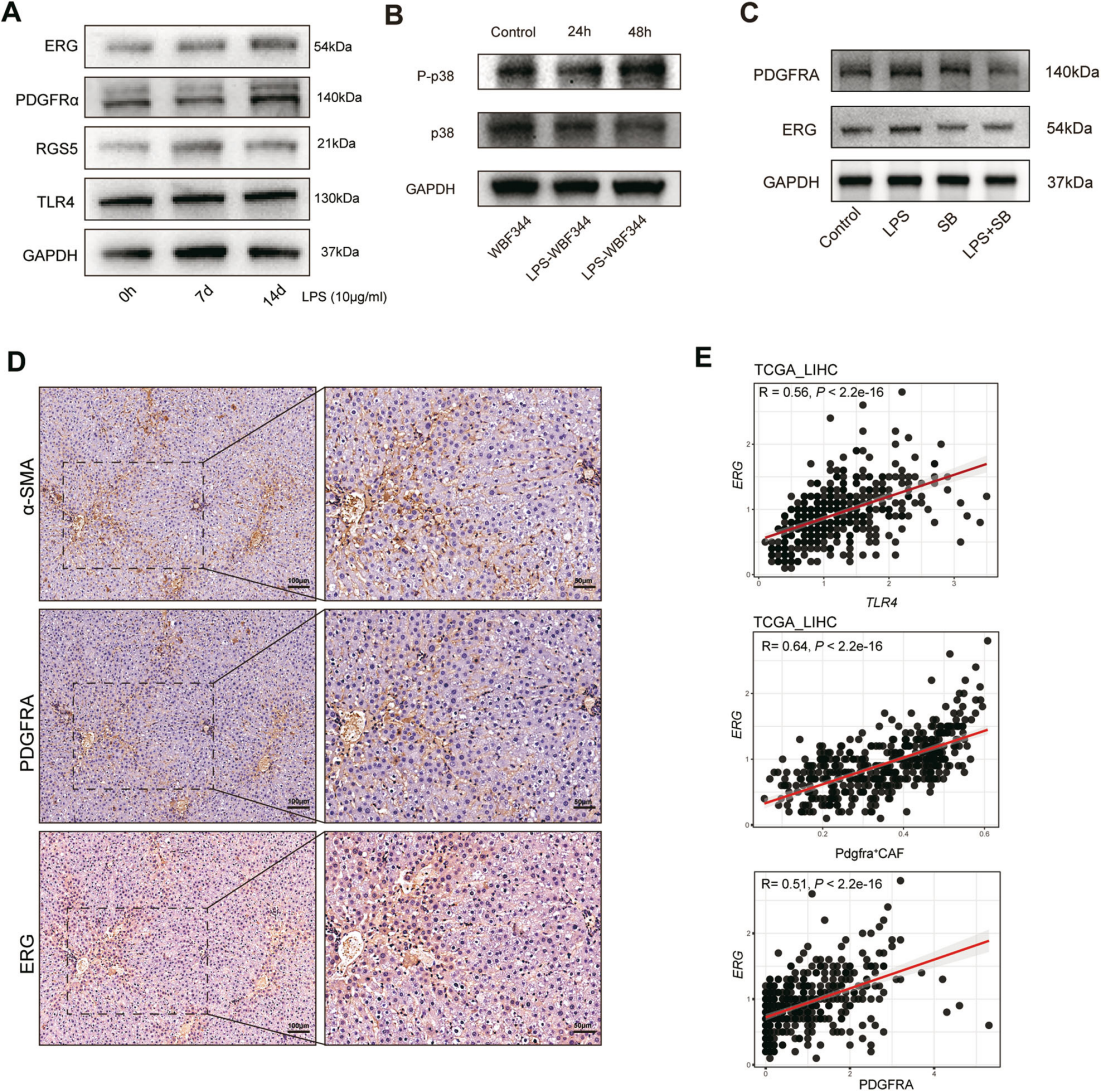
The TLR4-P38 MAPK signalling pathway contributed to the LPS-induced differentiation of HPCs into PDGFRα^*+*^ CAFs. **A** WBF344 cells were treated with LPS for 7 days or 14 days and TLR4, RGS5, PDGFRα and ERG expression was examined in each group by western blotting. GAPDH was used as the internal reference. **B** WBF344 cells were treated with LPS for 24 h or 48 h and the P38 MAPK and Pho-P38 MAPK levels in each group were examined by western blotting. GAPDH was used as the internal reference. **C** LPS and a P38 MAPK inhibitor were used to treat WBF344 cells and then the expression of ERG and PDGFRA was subsequently examined in each group by western blotting. **D** α-SMA, PDGFRα and ERG expression was detected by IHC in liver tissues from rats exposed to DEN for 12 weeks. **E** Correlation between TLR4 and ERG expression in TCGA-LIHC cohort.

TLR4 is one of the most important receptors for LPS, and we have shown the role of TLR4 in mediating the effect of LPS in HPCs [12]. The results revealed that TLR4 was highly expressed in WBF344 cells (Fig. 6A). As shown in Fig. S1G, GSEA revealed the upregulation of MAPK signalling pathways, such as the p38 MAPK pathway. Downstream of TLR4 in the MyD88-independent pathway, TLR4 recruits TRIF (TIR domain-containing adaptor inducing IFN-β), which activates the transcription factor interferon regulatory factor 3, to promote delayed P38 MAPK activation. P38 MAPK belongs to the mitogen-activated protein kinase (MAPK) family of stress-activated protein kinases. P38 MAPK plays a very important role in biological processes such as cellular stress, the inflammatory response, cell proliferation, differentiation, and apoptosis by phosphorylating downstream proteins. We validated the effect of P38 MAPK on the differentiation of HPCs and detected high levels of P38 MAPK and Pho-P38 MAPK in LPS-treated WBF344 cells (Fig. 6B). Furthermore in vitro experiments confirmed that inhibition of P38 MAPK notably suppressed the expression of ERG and the iCAF marker PDGFRA (Fig. 6C). ERG phosphorylation is mediated by mitogen-activated protein kinase signal-regulated kinase (MAPK) signalling, which promotes progenitor proliferation [17].

IHC staining also revealed a positive correlation between ERG and PDGFRα (Fig. 6D). Based on this analysis, we detected significant correlations between TLR4 and ERG expression in iCAFs derived from HPCs (*p* < 2.24e−16, *r* = 0.56), between ERG expression and PDGFRα^+^ CAFs (*p* = 2.24e−16, *r* = 0.64), and between ERG expression and PDGFRα expression (*p* < 2.24e−16, *r* = 0.51) in TCGA LIHC cohort (Fig. 6E). These results suggest that the TLR4-P38 MAPK signalling pathway contributes to the LPS-induced differentiation of HPCs into PDGFRα^+^ CAFs .

### ERG plays a key role in regulating the differentiation of HPCs towards PDGFRα^+^ CAFs in clinical human HCC samples

**Fig. 7:**
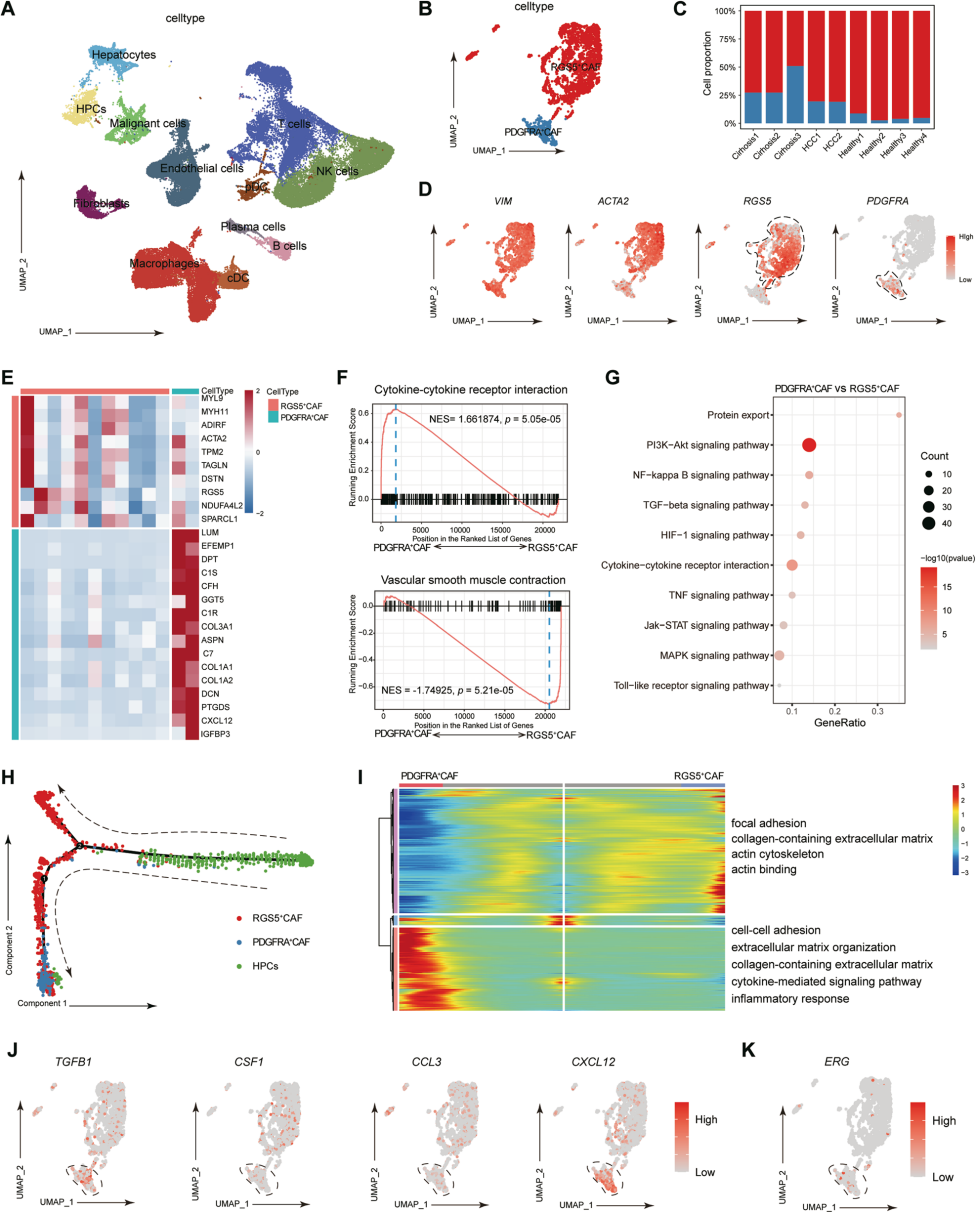
ERG plays a key role in regulating the differentiation of HPCs towards PDGFRα^+^ CAFs in clinical human HCC samples. **A** UMAP plot showing single cells profiled in the present work, colored according to major cell types. **B** UMAP plot showing CAFs coloured by subgroup markers. **C** Proportion of CAF subgroups in different patients. **D** UMAP plot showing representative scRNA-seq data for the CAF markers *VIM, ACTA2, RGS5* and *PDGFRA*. **E** Heatmap showing DEGs between the two CAF subgroups. **F** GSEA revealed the top enriched pathways in RGS5^+^ CAFs and PDGFRα^+^ CAFs. NES denotes normalized enrichment score. **G** Dot plot showing enriched KEGG pathways of upregulated pathways between PDGFRα^+^ CAFs and RGS5^+^ CAFs. **H** Trajectory of differentiation from HPCs into PDGFRα^+^ CAFs and *RGS5*^+^ CAFs, as predicted by Monocle 2. **I** Heatmap showing the enriched GO terms of upregulated genes between PDGFRα^+^ CAFs and RGS5^+^ CAFs. **J** UMAP plot showing the expression of *TGFβ, CSF1, CCL3* and *CXCL12* in PDGFRα^+^ CAFs. **K** UMAP plot showing the expression levels of *ERG* in human HCC.

We have demonstrated that ERG and PDGFRα^+^ CAFs play key roles in the immunosuppressive microenvironment during hepatocarcinogenesis in animal HCC models. Then, we investigated the relationship between ERG expression in HPCs and the aberrant differentiation of HPCs into PDGFRα^+^ CAFs as well as the prognosis of HCC patients. We performed scRNA sequencing of liver tumours from 2 HCC patients and downloaded liver scRNA sequencing data for healthy individuals (*n* = 4) and patients with liver cirrhosis(*n* = 3) from the GEO database (GSE136103 and GSEGSE149614, Fig. 7A). Subsets of CAFs derived from HPCs were identified as PDGFRα^+^ CAFs and RGS5^+^ CAFs (Fig. 7B). As we reported in a previous study (Fig. S1D, Supporting Information), the cell proportion of PDGFRα^+^ CAFs was high in the subset of patients with liver cirrhosis (before hepatocarcinogenesis) (Fig. 7C). The Expression of CAF markers was also upregulated in HPCs from cirrhosis and HCC tissues (Fig. 7E). GSEA of bulk RNA sequencing data also revealed the activation of PDGFRα^+^ CAFs and RGS5^+^ CAFs, and activated pathways included cytokine‒cytokine receptor interactions and vascular smooth muscle contraction (Fig. 7F). RGS5 and PDGFRα were expressed in HPCs from cirrhotic and HCC samples, which was consistent with the results observed in a rat HCC model. The enrichment analysis of DEGs revealed that PI3K-AKT, MAPK, and cytokine‒cytokine receptor interaction signalling were also activated in HPCs between cirrhotic and tumour tissue from clinical patients (Fig. 7G), which suggested that the signalling pathways mentioned above might contribute to the transformation of HPCs into CAFs. Pseudotime analysis also suggested that PDGFRα^+^ CAFs and RGS5^+^ CAFs from cirrhosis and HCC patients also diverged from HPCs, as we found in the rat HCC model (Fig. 7H). Changes in the expression profile were observed between RGS5^+^ CAFs and PDGFRα^+^ CAFs in the pseudotime trajectory, GO enrichment analyses of BP terms revealed that PDGFRα^+^ CAFs from patients with liver cirrhosis and HCC were enriched mainly in functions related to the inflammatory response. These results suggest that PDGFRα^+^ CAFs and RGS5^+^ CAFs might also be derived from HPCs in patients with liver cirrhosis and HCC patients. As shown in Fig. 7J, *TGFβ, CSF1, CCL3* and *CXCL12* were relatively upregulated in PDGFRα^+^ CAFs closely associated with the immunosuppressive microenvironment, as we observed in the rat HCC model. The transcription factor ERG was highly expressed in PDGFRα^+^ CAFs, which suggested that ERG mainly contributed to the differentiation of HPCs into PDGFRα^+^ CAFs (Fig. 7K).

## Discussion

Notably, recent studies have revealed a correlation between CAFs and immunosuppression, which warrants an investigation of the CAF-immune crosstalk. CAFs are among the components of the immunosuppressive TME [18]. A variety of mechanisms are reported to be involved in CAF-mediated modulation of HCC progression, including direct effects on cancer cells through the secretion of soluble factors and exosomes, as well as indirect effects through the remodelling of the extracellular matrix [19]. CAFs increase tumour cell malignancy and recruit immune cells to mediate immune escape [20, 21]. A recent study suggested that interleukin-6 (IL-6), derived from CAFs can recruit and regulate the survival, activation, and function of neutrophils via the signal transducer and activator of transcription 3-programmed cell death ligand 1 (STAT3-PDL1) signalling pathway. This pathway, in turn, contributes to the immune tolerance of HCC cells [22]. Furthermore, The CAFs derived from HCC cells also inactivate NK cells by secreting indoleamine 2,3-dioxygenase (IDO) and PGE2, thereby forming an appropriate immunotolerant niche and facilitating the progression of the disease [23]. In another study, CAFs secreted TGF-β, increasing the number of proliferating Tregs and accelerating HCC growth in the hepatic TME [24]. However, these studies cannot exclude other mechanisms through which CAF-immune crosstalk regulates hepatocarcinogenesis. In our study, we found that iCAFs might be derived from HPCs and participate in the construction of an ISME during hepatocarcinogenesis. Furthermore, iCAFs can recruit macrophages via several chemokines and polarize macrophages into the M2 phenotype, which has immunosuppressive properties.

HPCs normally reside in biliary ducts and can be activated by chronic liver damage [13, 14]. During acute liver injury, normal hepatocytes repair liver injury via proliferation and an increased cell volume [15]. However, the proliferative potential of liver cells is exhausted during chronic liver injury and HPCs are activated to repair liver injury through hepatotropic and biliary differentiation [16]. In our previous study, LPS induced HPCs to differentiate into myofibroblasts and HPC-derived myofibroblasts creates a tumour microenvironment and contributed to the proliferation and malignant transformation of HPCs, which eventually led to hepatocarcinogenesis. These results indicate that both CAFs and tumour cells are derived from HPCs [12]. Here, we applied a DEN-induced primary rat HCC model that can simulate damage-induced liver fibrosis, cirrhosis, and ultimately HCC well, which is similar to the pathogenesis of clinical HCC patients. We generated a comprehensive single-cell atlas of the liver of the HCC model to understand the cellular landscape during hepatocarcinogenesis. These results suggest that the differentiation of HPCs towards iCAFs potentially contributes to the formation of an ISME.

We explored the subgroups of CAFs derived from HPCs at different time points in a DEN-induced primary HCC model and validated them in a human single-cell RNA dataset. We identified a *Pdgfra*^**+**^ CAFs subset that was characterized as inflammatory cancer-associated fibroblasts (iCAFs) at 12 weeks in the HCC model. We isolated PDGFRα^**+**^ CAFs from primary HCC tissues from DEN-induced model rats at 8 and 12 weeks. The conditioned medium collected PDGFRα^+^ CAFs at 12 weeks secreted chemokines, including CCL3 and CXCL12, to recruit macrophages and produce cytokines including TGFβ, to polarize macrophages into the M2 phenotype, which has immunosuppressive properties. In vitro experiments also confirmed that when HPCs were treated with LPS for 14 days, HPCs may have differentiated into PDGFRα^+^ CAFs. Therefore, the results of this study suggested that HPC-derived *Pdgfra*^+^ CAFs by LPS might participate in the development of an ISME in HCC.

Macrophages, known as a heterogeneous cells, can be generally classified into two subtypes: termed classically activated macrophages (M1 or M1-like phenotype) and alternatively activated macrophages (M2 or M2-like phenotype) [25]. Macrophages within the tumours, known as tumour-associated macrophages (TAMs), are critical regulators of the immunosuppressive TME for immune escape and tumour development, and are involved in the formation of an ISME [26]. The majority of TAMs have the M2 phenotype and produce immunosuppressive factors such as TGFβ and IL-10 to support immunosuppressive cells such as myeloid-derived suppressor cells (MDSCs) and regulatory T cells (Tregs) [27]. We also found that PDGFRα^**+**^ CAFs were related to the recruitment of M2 macrophages. Therefore, we speculated that iCAFs might recruit M2 macrophages to construct the ISME of HCC.

TLR4 is one of the key receptors for LPS that plays an important role in mediating the function of LPS. As we have reported in previous studies, LPS not only leads to the differentiation of HPCs towards CAFs, but also plays a key role maintaining the stemness of cancer stem cells in HCC [12, 28]. The MAPK pathway, as one of the downstream signalling events mediated by TLR4, is significantly enriched in HPC-derived iCAFs as we reported in the human and rat scRNA-seq databases. Abnormal and excessive activation of the MAPK signalling pathway leads to the malignant transformation and evolution of tumour cells. Therefore, we inferred that the TLR4/MAPK signalling pathway leads to the malignant transformation of HPCs into CAFs.

Platelet-derived growth factor receptor alpha (PDGFRα), is a tyrosine kinase receptor that activates the PI3K/Akt, RAS/MAPK, and JAK/STAT pathways to promote cell proliferation and survival [29]. Abnormal activation of the PDGFRα pathway induces tumour neovascularization, which directly or indirectly promotes tumour cell proliferation and metastasis [30]. The results of our study indicated that the TLR4/P38 MAPK pathway promotes the aberrant differentiation of HPCs into iCAFs as well as the expression of PDGFRα.

We further investigated the potential molecular mechanisms of the LPS-induced differentiation of HPCs into PDGFRα^*+*^ CAFs. The results showed that LPS activated P38 MAPK and the downstream transcription factor ERG through TLR4, regulating the level of PDGFRα and ultimately inducing the aberrant differentiation of HPCs. In leukaemic cells, ERG phosphorylation is mediated by mitogen-activated protein kinase signal-regulated kinase (MAPK) signalling, which promotes progenitor proliferation and is a potential target for modulating ERG-driven transcriptional programs in leukaemia [17]. The ETS-1-related gene (ERG), located on chromosome 21q22, was first described in colorectal carcinoma cells by Reddy et al. [31]. ERG functions in cell proliferation, differentiation, inflammation, metastasis, haematopoiesis and immune development (especially for the maintenance of the haematopoiesis stem cell population), B-cell and megakaryocyte differentiation, and endothelial cell apoptosis [32]. ERG is one of the most consistently overexpressed oncogenes in malignant prostate cancer and is also implicated in other cancers, including Ewing’s sarcoma and leukaemia [33–35].

In this study, compared with that in the control group, the expression of ERG was significantly higher in PDGFRα^+^ CAFs. Once ERG expression was downregulated, the expression of PDGFRα in iCAFs stimulated with LPS decreased. Furthermore, hepatocarcinogenesis was also diminished at 8 weeks in DEN-induced rats treated with the ERG-knockout PDGFRα^+^ CAFs. Finally, we confirmed the relationships among the levels of EGR, PDGFRα and PDGFRα^+^ CAFs in the TCGA database. These results suggest that the expression of the transcription factor ERG plays a key role in mediating LPS-induced HPC differentiation into PDGFRα^+^ CAFs and the production of TGFβ, CSF1, CCL3 and CXCL12, which might be play key roles in the ISME of HCC.

In summary, we employed scRNA sequencing to explore subgroups of CAFs at different time points in a DEN-induced primary HCC model and validated the results in a human single-cell RNA dataset. We identified a CAF subset that was characterized as PDGFRα^+^ CAFs in the D12 HCC model. CAFs are a major component of the tumour microenvironment and play important roles in the growth, metastasis and therapeutic resistance of tumour cells. HCC cells may escape immune surveillance in the ISME, which is one of the reasons for the development of HCC tissues. Reversing ISME through treatments such as PD-1 therapy has become an important strategy in clinical tumour immunotherapy. Interestingly, on the one hand these PDGFRα^+^ CAFs that we identified in HCC tissue exhibit an immunosuppressive characteristics; on the other hand we find evidence that these PDGFRα^+^ CAFs may be derived from HPCs, which suggests that HPC-derived PDGFRα^+^ CAFs may participates in the construction of an ISME during hepatocarcinogenesis. Further mechanistic studies revealed that LPS activated P38 MAPK and the downstream transcription factor ERG through TLR4, promoting the differentiation of HPCs to PDGFRα^+^ CAFs, and that knocking down *Erg* in HPCs significantly inhibited the recruitment of TAMs in HCC tissue, thereby inhibiting the progression of HCC. Finally, the correlations of ERG, PDGFRα and HPC differentiation to PDGFRα^+^ CAFs were also confirmed in clinical samples. This study helps us to understand the mechanism underlying the formation of ISME of HCC, and then explores new strategies to improve the clinical effect of immunotherapy on HCC.

## Materials and methods

### Animal models and HCC tissues

Male SD rats (4–6 weeks old, weighing 160–180 g) were obtained from Shanghai Laboratory Animal Center (Shanghai, China), and were housed in a SPF animal facility. After a one-week acclimation period in the SPF facility, the rats were blindly randomly divided into 4 groups, with 6 rats in each group. All rats received DEN at a concentration of 95 mg L^−1^ in their drinking water. At D4, D8, D12, D16 time points, the rats were sacrificed to obtain liver samples.

HCC tissue specimens were obtained from 58 HCC patients who underwent hepatic resection at the Third Affiliated Hospital of Naval Medical University from 2000 to 2009. The clinical features are presented in Table 1. All the samples were subjected to immunofluorescence analysis. Freshly resected hepatobiliary tumours were collected from patients who were treated at the Third Affiliated Hospital of Naval Medical University after obtaining informed consent.

### Cell culture and treatment

WB-F344 cells were incubated in Dulbecco’s modified Eagle’s medium (DMEM, Gibco-BRL, Gaithersburg, MD, USA), containing 10% FBS and 1% penicillin and streptomycin, in a humidified atmosphere with 5% CO_2_ at 37 °C. WB-F344 cells were treated with LPS (Sigma, Saint Louis, MO, USA) at 10 μg/ml concentration. NR8383 cells were incubated in Ham’s F12K medium (Gibco, Gaithersburg, MD, USA) with 20% FBS and 1% penicillin and streptomycin, in a humidified atmosphere with 5% CO2 at 37 °C. All cells were obtained from The Chinese Academy of Sciences cell bank. All cells have been tested negative for mycoplasma contamination and were authenticated by STR profiling.

### Preparation of single-cell suspensions

Liver and tumour tissues were processed immediately after being obtained from the DEN -induced rat HCC primary model. Each sample was cut into small pieces (<1 mm), and the pieces were incubated with 1 mL of collagenase IV and 100 μL of DNase (Servicebio) for about15-30 min on a 37 °C shaker. Subsequently, 4 mL of DMEM was added to dilute the suspension, and 70 μm cell mesh was subsequently used to filter the suspension. After centrifugation at 250 × g for 5 min, the supernatant was discarded, and the cells were washed twice with PBS. Then, the cell pellet was resuspended in 1 mL of ice-cold red blood cell lysis buffer and incubated at 4 °C for 10 min. Next, 10 mL of ice-cold PBS was added to the tube, which was then centrifuged at 250 × *g* for 10 min. After the supernatant was decanted, the pellet was resuspended in 5 mL of PBS containing 0.04% BSA. Finally, 10 μL of the suspension was counted under a microscope. Trypan blue was used to quantify liver cells.

### Single-cell RNA sequencing

Single-cell RNA sequencing was performed by Shanghai NovelBio Co., Ltd. Chromium Single Cell 3′ Reagent v3 kits were used to prepare barcoded scRNA-seq libraries according to the manufacturer’s protocol. The cell suspension was loaded onto a chromium single-cell controller instrument (10× Genomics) to generate single-cell gel beads in the emulsion (GEMs). Approximately 12,000 cells were added to each channel, and the target cell recovery was estimated to be 8000 cells. After the generation of GEMs, reverse transcription reactions were performed to generate barcoded full-length cDNA. The emulsions were disrupted using the recovery agent, and then cDNA clean-up was subsequently performed with DynaBeads MyOne Silane Beads (Thermo Fisher Scientific). Next, cDNA were amplified by PCR for the appropriate number of cycles, which depended on the number of recovered cells. For single-cell RNA-seq library preparation, single-cell RNA-seq libraries were constructed using the Single Cell 30 Library Gel Bead Kit V2. Sequencing was performed on the Illumina HiSeq XTEN platform (Illumina, 150-bp paired-end protocol), according to the manufacturer’s protocol.

### Analysis of scRNA sequencing data

For all analyses, the rat genome (ensemble v93) was used as a reference. For quality control, three quality measurements were calculated, including the number of total genes and transcripts and the percentage of mitochondrial genes. Cells that expressed more than 25% mitochondrial genes and 40,000 transcripts or fewer than 600 genes were removed. The normalized and batch-corrected data were imported into Seurat (V4.0.4) for downstream analysis and visualization. Dimensionality reduction was performed via principal component analysis (PCA). Unsupervised cell clusters of the same major cell type were selected for uniform manifold approximation and projection (UMAP) analysis, graph-based clustering, and marker analysis to identify the cell subtypes. The marker genes were calculated using the Seurat package FindMarkers function with the Wilcoxon rank-sum test algorithm based on the following criteria: (1) logFC > 0.25; (2) *p* < 0.05; and 3) min.pct > 0.1.

The Seurat package FindMarkers function with the Wilcoxon rank sum test algorithm were used with the following criteria to identify DEGs between each group: (1) logFC > 0.25; (2) *p* < 0.05; and (3) min.pct > 0.1. KEGG and GO enrichment analyses of the functions of the DEGs were conducted.

A cell communication analysis was performed via CellPhoneDB [36], a public database of ligands, receptors, and their interactions, to identify cellular interactions. The membrane, secreted, and peripheral proteins of the clusters were annotated. The mean and cell communication significance (*p* < 0.05) were calculated based on the interactions, and the normalized cell matrix was obtained via Seurat normalization. The total number of ligand–receptor pairs between two clusters was obtained, and interactions were visualized as dot plots. Nichenet was utilized to better understand cell-to-cell interactions [37]. This analysis included many public databases (KEGG, ENCODE, and PhoshoSite) to track the receptor target in the provided dataset. Single-cell transcriptome data including liver tissues from healthy donors, patients with cirrhosis and patients with HCC, were obtained from the Gene Expression Omnibus (GEO; https://www.ncbi.nlm.nih.gov/geo/) database. The liver tissues of healthy donors were obtained from GSE136103 [38], as were the liver tissue cells of patients with cirrhosis. Liver tissue cells of patients with HCC were obtained from GSE149614 [39].

The cell lineage trajectory of HPCs was inferred by using the Monocle2 (V.2.18.0) R package while monocyte trajectories were learned from the explicit master graph of single-cell genomics data via reversed graph embedding to sort cells, thus solving complex biological processes robustly and accurately [40–42]. We used the “differentialGeneTest” function to derive DEGs from each cluster, and after constructing the cell trajectory, we detected DEGs in pseudotime. All the pseudotime-dependent genes were visualized via the plot pseudotime heatmap function with a CellDataSet object. Lineage trajectory plots and smooth expression curves based on CellDataSet were generated by plotting the cell trajectory and plotting_genes_in_pseudotime, respectively.

We used SCENIC in conjunction with the mm9 RcisTarget database to examine the roles of transcriptional regulators in HCC [43]. The unique molecular identifier (UMI) counts for all transcriptionally defined fibroblasts were provided as input. Based on these matrices, we constructed coexpression modules between transcription factors and potential target genes filtered by importance. Genes with significant motif enrichment were considered direct targets for a given transcription factor using the default parameters and defined as a regulon. Each regulon was then scored according to its active value using ranked genes. The cells were then reclustered using a matrix of regulon values, and the regulons were judged to be “active” in the cells according to the default threshold parameters [44].

### Immunohistochemistry and immunofluorescence staining

The sections were deparaffinized in xylene and rehydrated through a gradient of alcohol solutions. Endogenous peroxidase were then inactivated with 3% hydrogen peroxide at room temperature for 20 min (only for IHC). Next, the antigen retrieval was enhanced by autoclaving sections in 0.1 mol L^−1^ citrate buffer (pH 6.0) for 2 min. After washes with PBS, the sections were blocked with 3% BSA at 37 °C for 30 min. The sections were then incubated overnight at 4 °C with the primary antibodies. Subsequently, an HRP-conjugated goat antibody and DAB (GeneTech, Shanghai, China) or fluorescent dye-labelled secondary antibodies were used. Images were captured with a microscope. At least three random areas per slide were selected to count the number of positively stained cells. IHC and IF analyses were performed using the following antibodies: alpha SMA (diluted 1:200, ab7817, Abcam, Cambridge, UK), PDGFR alpha (diluted 1:200, ab203491, Abcam, Cambridge, UK), RGS5 (diluted 1:200, DF4417, Affinity, Jiangsu, China), PLA2G2A(diluted 1:200, DF6366, Affinity, Jiangsu, China), ERG (diluted 1:200, ab92513, Abcam, Cambridge, UK), CD68 (diluted 1:200, ab125212, Abcam, Cambridge, UK), CD163 (diluted 1:400, ab182422, Abcam, Cambridge, UK), and iNOS (diluted 1:1000, ab283655, Abcam, Cambridge, UK).

### Real-time PCR

Total RNA was extracted using a HiPure Total RNA Plus Micro Kit (Magen, China) and reverse transcribed into cDNA using a Bestar qPCR RT Kit with a total reaction volume of 20 μL. qPCR was conducted using a Bestar one-step RT qPCR kit (Sybr Green) (DBI, China) according to the manufacturer’s instructions. The running parameters for qPCR were set as follows: 95 °C for 1 min (predenaturation), followed by 40 cycles of 95 °C for 15 s (denaturation), 60 °C for 30 s (annealing), and 72 °C for 15 s (extension). GAPDH was used as an internal reference.

### Bulk RNA sequencing analysis

Total RNA was extracted from each tissue sample using TRIzol (Life Technologies, Grand Island, NY, USA), according to the protocol provided by the manufacturer. Five micrograms of RNA from each sample were individually used for the construction of transcriptome libraries, using the Illumina TruSeq RNA Sample Preparation Kit (Illumina, San Diego, CA, USA), and sequenced using the Illumina HiSeq 2000 platform according to the manufacturer’s instructions. Q20 was used as a quality control standard to filter raw reads. After the low-quality reads were filtered, the adaptors of the high-quality reads were removed, and the clean reads were subsequently aligned to the rat genome, using the UCSC rat reference genome (http://genome.ucsc.edu). The fragments per kilobase of exon model per million mapped reads (FPKM) values were calculated according to the counts and lengths of genes. The DGEs with a fold change (FC) ≤ 0.5 or FC ≥ 2 and *p* < 0.05 were selected. For gene GSEA analysis, normalized values of RNA-seq data (FPKM) were rank-ordered by the fold change as input. The analysis was performed using GSEA (version: 4.3.3, https://www.gsea-msigdb.org/gsea/index.jsp) software. Sequencing was performed by Biomarker (Beijing, China).

Transcriptome datasets were also collected from The Cancer Genome Atlas (TCGA-LIHC; https://portal.gdc.cancer.gov/). The transcriptomes of 52 HCC-adjacent nontumor tissues were obtained from the GSE76427 [45].

### Olink proximity extension assay

The Olink proximity extension assay (Olink Proteomics AB, Uppsala, Sweden) was used to perform the proteomic analysis between CAF-CM and normal medium. The CAF-CM and normal medium were analysed using the Olink Inflammatory and Oncology panels. The Olink platform is a multiplex DNA-coupled immunoassay-based targeted proteomic approach in which each target protein is detected by a pair of unique oligonucleotides-labelled antibodies. When the antibody probes bind to their targeted protein, they form a target sequence that is later quantified by RT-PCR. Counts of known sequences are thereafter translated into normalized protein expression (NPX, which is an arbitrary unit on a log2-scale where a high value corresponds to high protein expression) units through a quality control and normalization process developed and provided by Olink. The data were quality controlled and normalized using an internal extension control and an interplate control to adjust for intra- and interrun variation.

### Isolation of CAFs

CAFs were isolated from HCC tissues from rats exposed to DEN for 12 weeks. Fresh liver tissues were washed with phosphate buffer and minced into small pieces (<1 mm^3^). The small tissues were attached to the cell culture dishes and treated with Dulbecco’s modified Eagle’s medium (DMEM medium, Gibco, USA), supplemented with 10% foetal bovine serum (FBS; Gibco, USA), 50 U/mol penicillin (Gibco, USA) and 50 mg/ml streptomycin (Gibco, USA). The DMEM medium was changed every two days. After digestion with trypsin, the fibroblasts extending from the liver tissue were transferred to the dish, followed by incubation in fresh medium to facilitate attachment of the isolated fibroblasts to the dish. The cells were maintained in a complete medium at 37 °C in a humidified incubator with 5% CO_2_ and 21% O_2_. CAF subgroup were sorted by flow cytometry. Cells and conditioned medium (CM) were collected for further experiments.

### Gene silencing mediated by shRNAs

For ERG interference, three shRNA candidates with ERG target sequences were designed by Hanbio (Shanghai, China). A scrambled shRNA served as the negative control. The 20 µM shRNA was diluted to 100 nM in medium. Then, the appropriate RNA mixture was added to form the transfection complexes and incubated for 15 min. The medium was replaced, and fresh medium containing the shRNA was added to cells for transfection. After 48 h, whether the target gene was pulled down was determined via real-time PCR (RT-PCR) and western blotting (WB).

### Flow cytometry

A total of 20 μl of anti-α-SMA antibody was added to 100 μl of the cell suspension (1 × 10^6^ cells/ml), the mixture was incubated at 4 °C for 30 min, and further stained with a secondary Alexa Flour 488-conjugated goat anti-rabbit IgG (Invitrogen, Carlsbad, CA, USA) antibody at 4 °C for 30 min. The Alexa Flour 488-conjugated isotype IgG antibody was used as a negative control. The stained cells were analysed on a FACS flow cytometer (Becton Dickinson, San Jose, CA, USA).

### Western blotting and gel electrophoresis

For western blot assays, the samples were lysed with radioimmunoprecipitation assay lysis buffer. Then, 10–25 µg protein samples were subjected to SDS‒PAGE (Bio-Rad, Hercules, CA, USA) on a 4–20% Bis-Tris SurePAGE gel (GenScript, Nanjing, CN). Proteins were transferred to polyvinyl difluoride membranes (Merck Millipore, Darmstadt, Germany) were used to transfer the protein. Next, the membranes were incubated with primary antibodies including anti-alpha SMA(diluted 1:1000, Abcam, Cambridge, UK), anti-ERG (diluted 1:1000, Abcam, Cambridge, UK), anti-PDGFR alpha (diluted 1:2000, Abcam, Cambridge, UK), anti-phospho-p38 MAPK (diluted 1:1000, Cell Signaling Technology, Beverly, MA, USA), anti-p38 MAPK (diluted 1:1000, Cell Signaling Technology, Beverly, MA, USA), anti-TLR4 (diluted 1:1000, Abcam, Cambridge, UK), anti-RGS5 (diluted 1:1000, Affinity, Jiangsu, China), anti-GAPDH (diluted 1:5000, Bioworld), at 4 °C for overnight, followed by an incubation with secondary antibodies (diluted 1:3000, ServiceBio, China) for 1.5 h. The samples were visualized with enhanced chemiluminescence detection reagents (GE HealthCare, Chicago, IL, USA). For gel electrophoresis, 1–5 μg of each DNA samples was loaded onto a 2% agarose gel, followed by electrophoresis at 160 V for 30 min in 1× TBE buffer. The gel was stained with ethidium bromide staining and visualized with a gel imaging system (Syngene, Frederick, MD, USA).

### Statistical analysis

All the experiments were performed at least three times. Analysis of variance was performed using GraphPad Prism 9.0 (GraphPad Software). The quantitative data are presented as the means ± SD, for each experiment. The significance of differences between groups was determined using Student’s *t*-test. Clinical data were analysed using SPSS 20.0 for Windows (SPSS Inc., Chicago, IL, USA); Pearson’s correlation coefficients were calculated to determine correlations between continuous normally distributed variables. Kaplan–Meier analysis was used to determine the survival duration. The log-rank test was performed to compare survival durations between patients in each group. Statistical significance is indicated by *p < 0.05, **p < 0.01, ***p < 0.001, and ****p < 0.0001.

## Supplementary information

Supplementary Table

Supplementary figure

Supplementary Legend

original data

## Data Availability

The single-cell RNA sequencing data generated in this study are available at the Gene Expression Omnibus (GEO, https://www.ncbi.nlm.nih.gov/geo/,GSE218561), RNA sequencing data are available from the corresponding author on reasonable request. Other relevant data are within the manuscript and its Additional files.
