# Supplementary material for: ERG mediates the differentiation of hepatic progenitor cells towards immunosuppressive PDGFRα+ cancer-associated fibroblasts during hepatocarcinogenesis
Source: Cell Death Dis. 2025 Jan 18;16(1):26. doi: 10.1038/s41419-024-07270-9 (PMC11743139; doi:10.1038/s41419-024-07270-9)
Supplement: Supplementary file 1 — Supplementary Table [file 41419_2024_7270_MOESM1_ESM.docx]

**Supplementary Table 1 The primer sequences of qRT-PCR**

| Target | Forward Sequence  (5’-3’) | Reverse Sequence  (5’-3’) | Size (bp) |
| --- | --- | --- | --- |
| Rat-ERG | CACCAGTAGTCG  CCTTGCTAATC | CGCCGAGCCAC  CTCATCC | 156 |

**Supplementary Table 2 siRNA&shRNA sequences**

| siRNA1 | GAGCAAGGACCAGTCACTATTTGAG |
| --- | --- |
| shRNA1 | Top strand:  GATCCGAGCAAGGACCAGTCACTATTTGAGCTCGAGCTCAAATAGTGACTGGTCCTTGCTCTTTTTTG |
|  | Bottom strand:  AATTCAAAAAAGAGCAAGGACCAGTCACTATTTGAGCTCGAGCTCAAATAGTGACTGGTCCTTGCTCG |
| siRNA2 | GATACTGTGGGAATGAGCTACGGCA |
| shRNA2 | Top strand:  GATCCGATACTGTGGGAATGAGCTACGGCACTCGAGTGCCGTAGCTCATTCCCACAGTATCTTTTTTG |
|  | Bottom strand:  AATTCAAAAAAGATACTGTGGGAATGAGCTACGGCACTCGAGTGCCGTAGCTCATTCCCACAGTATCG |
| siRNA3 | CCTCCTAGACGTGGACGTCTTATTA |
| shRNA3 | Top strand:  GATCCGCCTCCTAGACGTGGACGTCTTATTACTCGAGTAATAAGACGTCCACGTCTAGGAGGTTTTTTG |
|  | Bottom strand:  AATTCAAAAAACCTCCTAGACGTGGACGTCTTATTACTCGAGTAATAAGACGTCCACGTCTAGGAGGCG |
