# Supplementary figures and images for: ERG mediates the differentiation of hepatic progenitor cells towards immunosuppressive PDGFRα+ cancer-associated fibroblasts during hepatocarcinogenesis
Source: Cell Death Dis. 2025 Jan 18;16(1):26. doi: 10.1038/s41419-024-07270-9 (PMC11743139; doi:10.1038/s41419-024-07270-9)

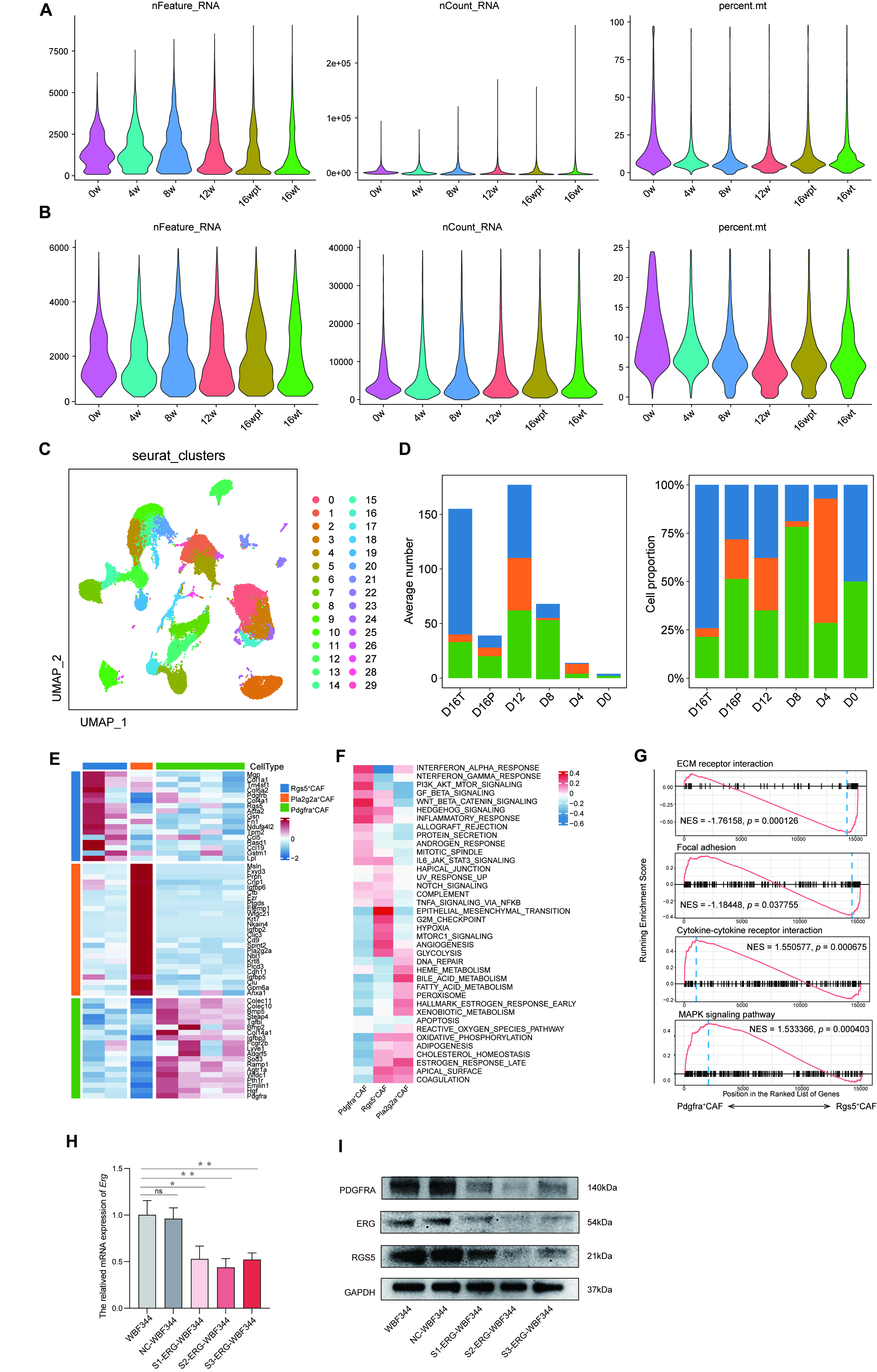

Supplement: Supplementary file 2 — Supplementary figure [file 41419_2024_7270_MOESM2_ESM.tif]

Figure 5

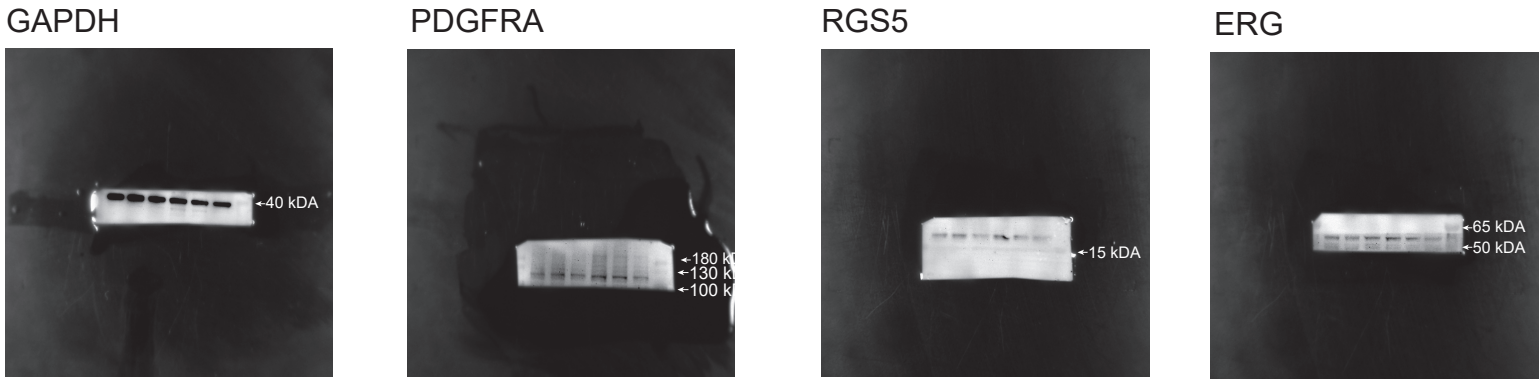

Figure 6B

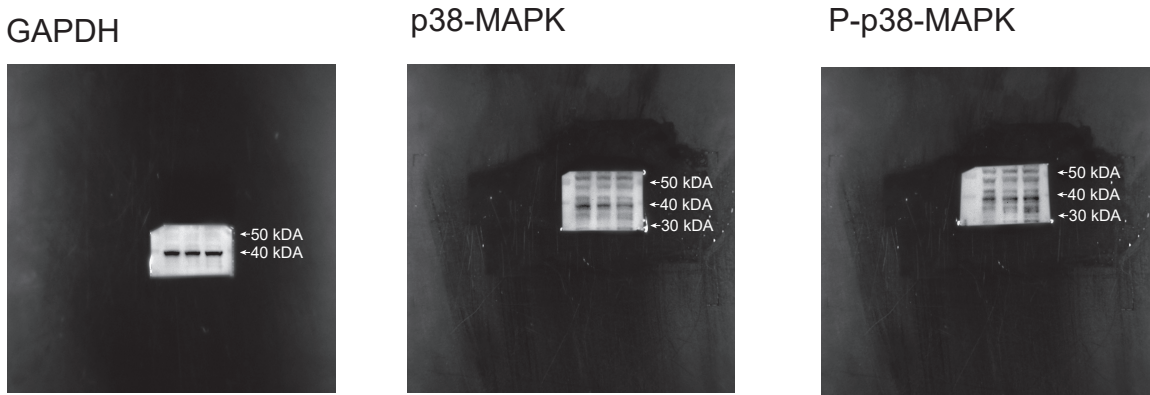

Figure 6C

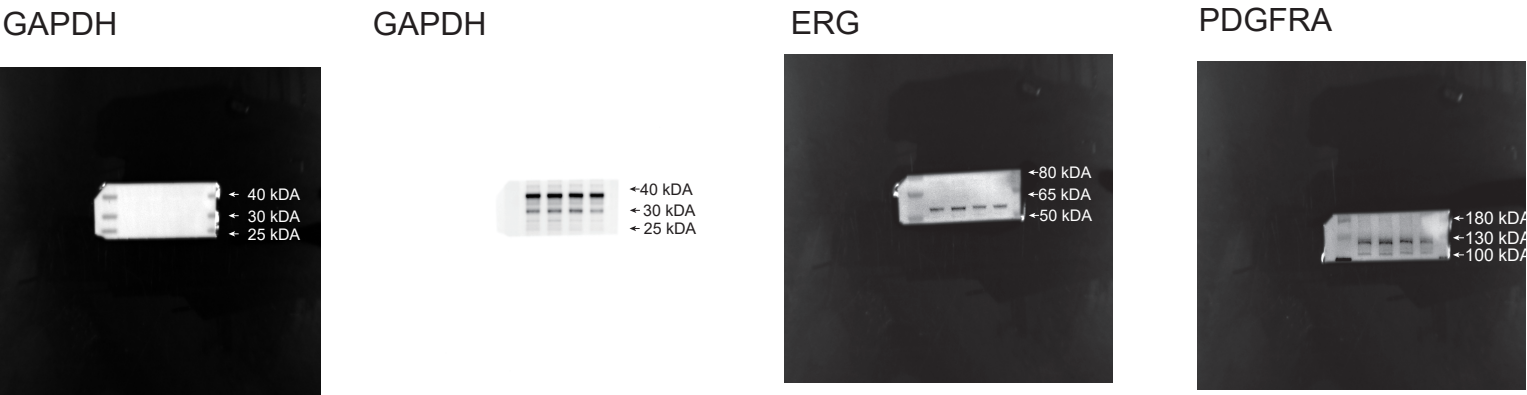

Figure 6A

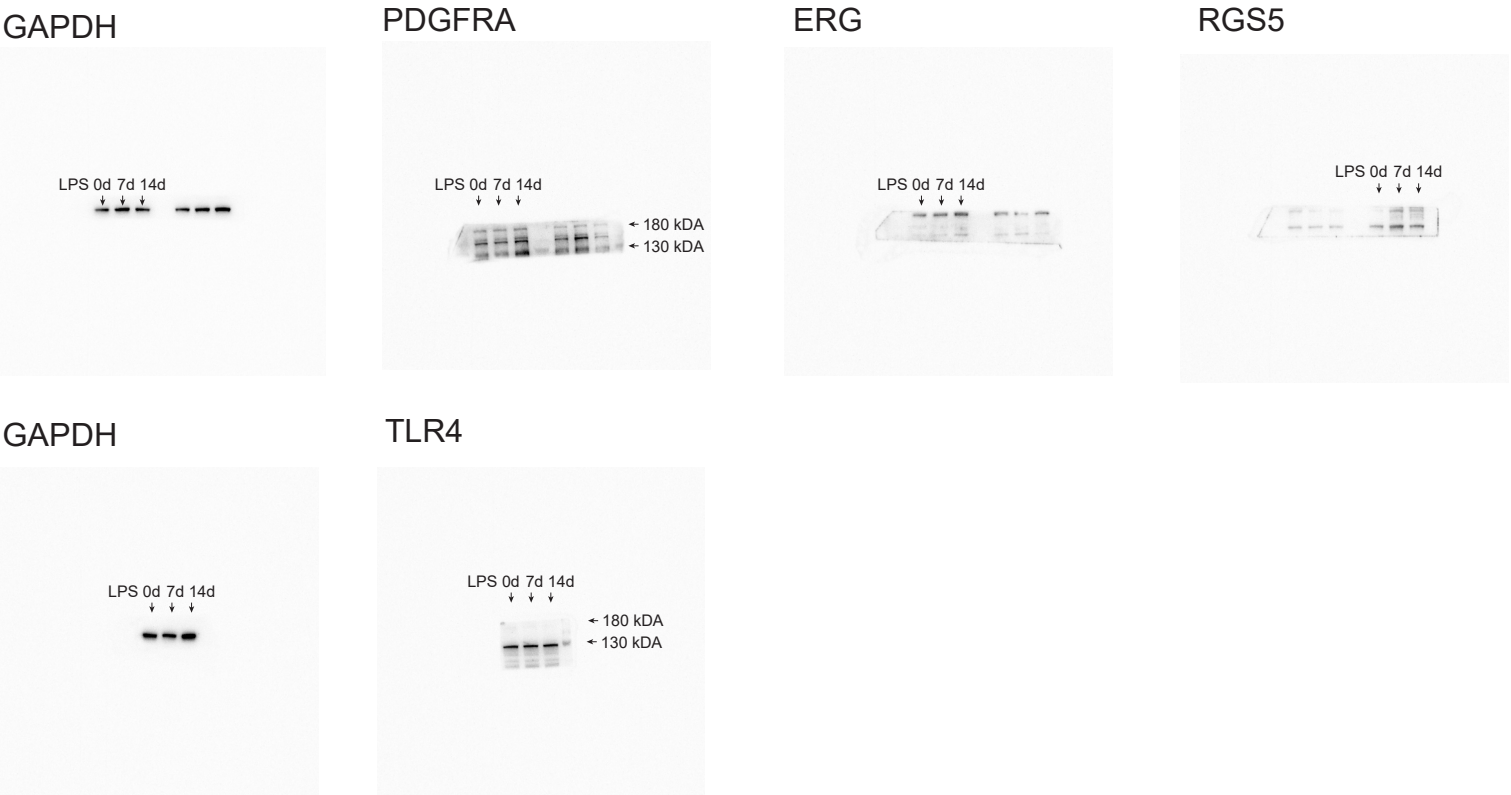

Supplement: Supplementary file 4 — original data [file 41419_2024_7270_MOESM4_ESM.pdf]
