# Supplementary material for: ERG mediates the differentiation of hepatic progenitor cells towards immunosuppressive PDGFRα+ cancer-associated fibroblasts during hepatocarcinogenesis
Source: Cell Death Dis. 2025 Jan 18;16(1):26. doi: 10.1038/s41419-024-07270-9 (PMC11743139; doi:10.1038/s41419-024-07270-9)
Supplement: Supplementary file 3 — Supplementary Legend [file 41419_2024_7270_MOESM3_ESM.docx]

**Supplementary Figure 1**

1. Violin plot (Vln plot) showing nFeature_RNA, nCount_RNA and percent.mt in the scRNA-seq data before quality control. **(B)** Vln plot showing nFeature_RNA, nCount_RNA and percent.mt in the scRNA sequencing data after quality control (nFeature_RNA≦6000, nCount_RNA≦40000, percent.mt≦25). **(C)** T-distributed stochastic neighbour embedding (t-SNE) plot showing the clustering results for single cells from DEN-treated rat livers. **(D)** Percentage distribution of cell types in the six DEN-induced rat liver samples. **(E)** Heatmap showing DEGs between the three CAF subgroups. **(F)** Heatmap showing differences in pathway activities per cell as scored by GSVA between different CAF subgroups. The *t* values from a linears model are shown. **(G)** GSEA revealed the top enriched pathways in PDGFRα^+^ CAFs and Rgs5^+^ CAFs). **(H)** Real-time PCR was used to detect ERG expression in WBF344 cells treated with shRNA-ERG. **(I)** ERG expression in WBF344 cells was inhibited by shRNA-*Erg* and the levels of ERG, PDGFRα and RGS5 were subsequently examined in each group by western blotting.
